# Editorial: Informal learning through work

**DOI:** 10.3389/fpsyg.2023.1156141

**Published:** 2023-06-13

**Authors:** Dominik E. Froehlich, Gerhard Messmann, Isabel Raemdonck

**Affiliations:** ^1^University of Vienna, Vienna, Austria; ^2^Faculty of Human Sciences, University of Regensburg, Regensburg, Germany; ^3^Université Catholique de Louvain, Walloon Brabant, Belgium

**Keywords:** informal learning, workplace learning, reflection, proactive behavior, learning from others, relational learning

Informal learning through work is learner initiated, happens outside formal training and is practice-based (Noe et al., 2013). In addition, informal learning may also take place while learners are participating in a purposefully designed formal learning environment (Poell, 2017). And in a similar vein, informal learning activities at work may represent a work-integrated continuation of formal learning activities. The interest in the concept of informal learning through work, and the range of topics that are being investigated, has increased vastly in the last decade. This Research Topic illustrates this development by focusing on a variety of manifestations of informal learning and a range of current topics in the field of learning in organizations and work-related learning (see Table 1). The seven studies cover diverse populations across functions and in seven countries, and are based on quantitative, qualitative, and mixed data.

**Table 1:** Studies of this Research Topic categorized by topic, population, and data.

**Article**	**Topic**	**Population**	**Data**
Amenduni et al.	Disruptive changes as triggers for workplace learning	Employees, employers, and adult educators in Italy, Switzerland, and Finland	Mixed
Crans et al.	Leadership influence on feedback-seeking	Employees in the Netherlands and Germany	Mixed
Hommel et al.	Multifaceted model of reflection	Teachers in Germany	Conceptual, qualitative
Kang	Power struggles as barriers to informal learning and innovation in teams	Work teams in Korea	Quantitative
Leiß et al.	Employees' problem solving; electronic support systems as triggers for informal learning	(HR) employees in Germany	Quantitative
Wang et al.	Organizational unlearning as facilitator of innovation performance	Managers in China	Quantitative
Wuytens et al.	Tacit entrepreneurial knowledge	Entrepreneurs	Conceptual, mixed

These seven studies not only help in reflecting the state-of-the-art of research on informal learning, but also provide a basis for further discussing themes for future research. In this editorial, we focus on three themes: *Reflection and learning from experience, learning from others*, and *proactivity in organizations*.

The first theme addresses the role of reflection for professional performance and development. Reflection refers to cognitive activities employees carry out to gain a better understanding of their work. Various objects of reflection may be discerned, including work tasks, the context, employees' perceived competences and their professional self-concept (Messmann, 2022). Reflection enables employees to make use of their work experience and make it accessible for future actions (e.g., Kolodner, 1992). Thus, reflection is an integral element of informal learning as it enables employees to connect past work outcomes to current work tasks, and current work tasks and outcomes to future work tasks. Likewise, reflection helps employees to self-regulate their work processes by linking goals, plans, actions, and outcomes of their professional performance. Finally, reflection is essential for integrating implicit, episodic aspects of professional knowledge (cf. Wuytens et al., who highlight the importance of tacit knowledge for entrepreneurial performance) with explicit, propositional aspects of professional knowledge as well as for combining one's own insights with the perspectives of peers, superiors, or professional communities. This multifaceted character of reflection is illustrated in the conceptual analysis by Hommel et al. who provide a guide for future research by integrating different objects and levels; triggers, facilitators, and outcomes; and accompanying emotional, motivational, and cognitive processes of reflection in one model.

This leads to the second theme: learning from others. The articles in this Research Topic highlight the considerable effect of others in the learning process. Crans et al. and Leiß et al. note the importance of asking colleagues for a consultation or feedback, respectively; Hommel et al. discuss the perspective of social relatedness for reflection, while Kang shows how negative social interactions may hamper learning. This echoes findings of previous research, for example, regarding the positive effects of learning from others on careers (Froehlich et al., 2019), mental health (Beausaert et al., 2021), or innovation processes (Froehlich and Messmann, 2017). But these relational questions also raise methodological issues (Pantić et al., 2022) and conceptual questions (Coppe et al., 2022) to be addressed in future research.

The third theme highlights the role of transformations in the world of work as triggers for informal learning and proactivity. Effects of disruptive changes in organizations and entire industries on informal learning are, for instance, explored by Amenduni et al.. Within such dynamic work environments, it is expected of professionals to not only possess efficient routines, but to proactively adapt to changes of work tasks and the surrounding context (Schwartz et al., 2005). Such proactive responses to work-related changes can manifest in various ways, depending on the severity of the changes. For instance, if employees struggle with a work task, the reason may simply be a lack of information. In this case, doing a quick search on the internet or asking a more experienced colleague for advice may resolve the problem. Crans et al. tackle this topic by exploring the relationship between leadership and feedback-seeking. But the problem may also be more fundamental. Then a new solution needs to be developed that innovates underlying assumptions, core elements, and/or goals of existing work processes. This issue is addressed in the studies by Kang and by Wang et al. who explore innovation processes at team and organizational level, respectively. Both manifestations of proactivity have in common that besides aiming at an immediate benefit for work processes, the work environment, or the entire organization, they contain an inherent learning potential which can contribute to employees' professional development.

The current Research Topic features three current and important avenues that help advance the field of informal learning through work by asking conceptual and methodological questions. The questions raised in the current contributions furthermore point to a much greater breadth of topics and contexts and conceptual variations in the field of informal learning through work which are unified by the idea that work and learning represent two sides of the same coin.

## Author contributions

All authors listed have made a substantial, direct, and intellectual contribution to the work and approved it for publication.
